# Youth‐inclusive framework for a sustainable future in planetary health action: A conference summary

**DOI:** 10.1002/puh2.81

**Published:** 2023-04-20

**Authors:** Adriana Viola Miranda, Katherine Feng, Ave Põld, Muhammed Yaseen Tagari, Sherly Meilianti, Shahd Sharief, Elaine Tan Su Yin, Rahmot Afolabi

**Affiliations:** ^1^ GHWN Youth Hub Geneva Switzerland; ^2^ Global Health Focus Asia Bandung Indonesia; ^3^ World Congress of Chiropractic Students Toronto Canada; ^4^ International Pharmaceutical Federation The Hague The Netherlands; ^5^ Sudan Medical Specialization Board (EDC, TDD) Khartoum Sudan; ^6^ World Youth Alliance Nairobi Kenya

**Keywords:** climate change, competency‐based education, digital health, governance, health workforce, planetary health, research, youth

## Abstract

The multisectoral nature of emerging global health threats, such as climate change, antimicrobial resistance, and food security, requires holistic solutions. Planetary health, which encompasses the relationship between human systems– political, economic, and social—and Earth's natural systems, is an emerging holistic approach used globally. The Global Health Workforce Network (GHWN) Youth Hub Virtual Conference 2022 was held on 9–10 September 2022 to discuss the role of youth in advancing planetary health. This conference summary introduces a novel youth‐inclusive planetary health framework and describes the current implementation landscape and challenges of planetary health. Based on literature review and discussions at the conference, we identified four supporting pillars of youth‐inclusive planetary health approaches: governance, competency‐based education, research, and digital health. Considering the increasing number of youth in the global health workforce and their huge interests in community participation, meaningful youth engagement should be prioritized to ensure the sustainability of these approaches.

## INTRODUCTION

Human actions have consequences on the health of the world. For example, climate‐related disruptions induced by anthropogenic activities, such as rapid urbanization, deforestation, transportation and pollution, are causing irreversible consequences on human and animal health [1]. It is reported that 58% of known infectious diseases can be aggravated by climate hazards [2]. The World Health Organization (WHO) estimates that between 2030 and 2050, climate change will cause 250,000 additional deaths and USD 2–4 billion direct health damage costs [3].

Other human‐induced disruptions have also led to negative health consequences. Each year, more than 10 million hectares of agricultural land are lost to degradation, increasing the risk of global hunger [4]. Antimicrobial resistance (AMR), which is estimated to cause 10 million annual deaths by 2050, is highly related to various Earth systems as resistant bacteria are known to be harbored in various ecosystem settings, from freshwater, soil, and meat to animal manure. AMR also leads to the production of toxic gasses and the dissemination of resistant genes to the Earth's critical zone (the outer layer of the Earth), which may cause further health disruptions [5]. These adverse outcomes have a heavier impact on many lower‐income countries and the most vulnerable groups of society, where underlying inequalities and weaknesses have been further exposed and exacerbated by the COVID‐19 pandemic. Each individual in lower‐income countries loses fifteen times the amount of healthy life years compared to higher‐income countries due to environmental exposures [6].

With these growing health and environmental challenges, investments are needed now more than ever to strengthen the resilience of health systems against natural and man‐made disasters. Implementing a holistic, interdisciplinary approach is, therefore, essential. Planetary health, defined as a field focusing on “analyzing and addressing the impacts of human disruptions to Earth's natural systems on human health and all life on earth,” could be the key to a sustainable and equitable future in global health and universal health coverage [1, 6]. Equipping the health care workforce with planetary health‐related skills is crucial in advancing the field. Investing in youth is particularly important, as youth is a primary stakeholder in the health care workforce. In 2030, youth will fill most of the >120 million jobs that will be created in the health care sector [7, 8]. This article describes the current nature of youth involvement in the planetary health landscape and proposes a new framework which emphasizes a meaningful youth engagement. Of the few frameworks published on planetary health, this is the first to have a specific focus on integrating youth into core action strategies.

## GHWN YOUTH HUB VIRTUAL CONFERENCE 2022

**FIGURE 1:**
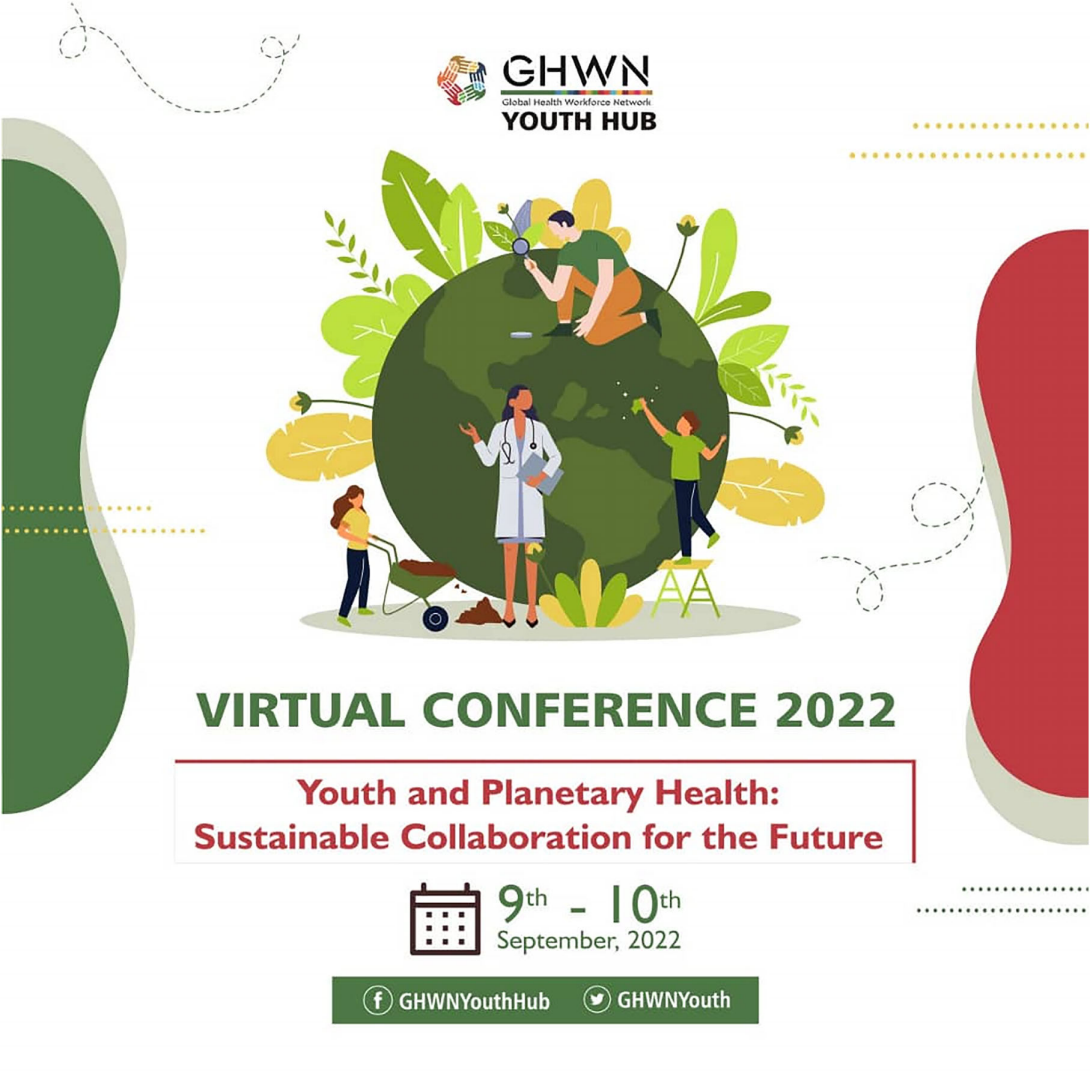
Global Health Workforce Network (GHWN) Youth Hub Virtual Conference 2022 poster.

This paper is a proceeding of the Global Health Workforce Network (GHWN) Youth Hub Virtual Conference 2022: “Youth and Planetary Health: Sustainable Collaboration for the Future” (Figure 1). The conference was held on 9–10 September 2022 by the GHWN Youth Hub, a youth‐focused, interprofessional community of practice hosted by the WHO and GHWN. It held 11 thematic plenary sessions and interactive discussions between youth health and care workers and planetary health experts from the WHO, World Bank, Food and Agriculture Organization (FAO), World Organization of Animal Health (WOAH), Africa Center for Disease Control and Prevention (Africa CDC), and among others. A hackathon called “YHack22” was also held in conjunction with the conference, serving as a platform for youth to brainstorm tangible planetary health solutions, increase entrepreneurial competencies, and establish connections with other youth in this space. The conference was attended by 210 youth from 53 countries. They came from various health care backgrounds, such as medicine, veterinary medicine, nursing, pharmacy, public health, engineering, among others. The results of the interactive discussions and hackathon were collated to be a reference for this proceeding.

## THE CONCEPT OF PLANETARY HEALTH

There are several holistic approaches that focus on the human–animal–environmental interface within the health sector. Some of the most influential concepts include One Health, EcoHealth, and the more recently developed planetary health. Planetary health was first introduced by the Rockefeller Foundation–Lancet Commission on Planetary Health as “the achievement of the highest attainable standard of health, well‐being, and equity worldwide through judicious attention to the human systems—political, economic, and social—that shape the future of humanity and the Earth's natural systems that define the safe environmental limits within which humanity can flourish” [6]. This definition is further translated by the Planetary Health Alliance as a solution‐driven, transdisciplinary field addressing the impacts of human disruptions to the Earth's natural systems on the health of humans and other organisms [1]. It is argued that planetary health provides cohesion to the other holistic approaches as it explicitly incorporates social science as well as behavioral aspects of health and governance [9]. The scope of planetary health is not limited to climate change, but also pollutants, degradation of marine systems, shortages of arable land and freshwaters, changes in land use, and other environmental changes [1].

## YOUTH‐INCLUSIVE PLANETARY HEALTH FRAMEWORK

**FIGURE 2:**
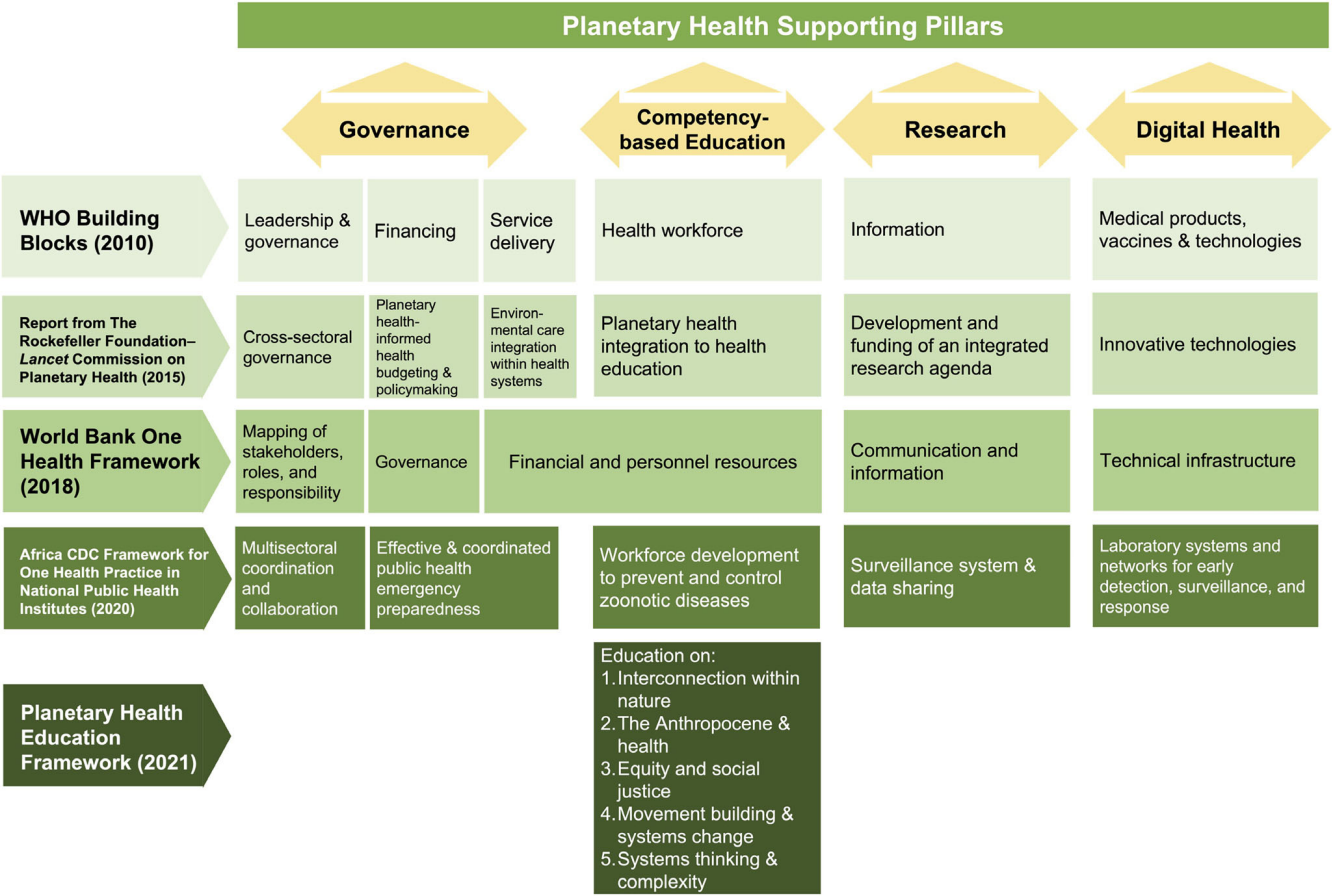
Mapping of the supporting pillars of planetary health. Four supporting pillars were identified based on the themes covered by the included frameworks.

To understand the current landscape of planetary health approaches, we conducted a literature review that included frameworks analyzing pillars on planetary health or similar holistic approaches (e.g., One Health). Based on the literature review and discussions at the GHWN Youth Hub Virtual Conference 2022, the GHWN Youth Hub identified four supporting pillars for advancing planetary health: governance, competency‐based education, research, and digital health. These areas were selected as they appear in relevant frameworks, including the WHO health system building blocks [10], a report from The Rockefeller Foundation–Lancet Commission on Planetary Health [6], the World Bank One Health Framework [11], the Africa CDC Framework for One Health Practice in National Public Health Institutes [12], and the Planetary Health Education Framework [13] (Figure 2).

We noted that in these frameworks, youth inclusivity is requires a thorough consideration. Youth are crucial in planetary health operationalization as they are among the most impacted by environmental issues. A 2021 UNICEF report found that approximately 1 billion (45.4% of global children population) live in one of the 33 countries marked with extremely high climate health risks [14]. Furthermore, UNICEF's flagship digital platform for engaging with youth, the U‐Report, identified in its latest poll that two out of five young people globally are reconsidering starting families due to climate change [15]. On the other hand, the annual volunteering rate among the population aged 15 and above sits at 57.6%, with youth being among the most active [16]. A global survey found that 75% of respondents agreed that engaging youth in the field of climate and health provides access to fresh perspectives and unique skills [17]. This highlights the huge potential of youth engagement in planetary health, where they have the power to build movements and coalitions to address gaps in climate and other environmental issues.

**FIGURE 3:**
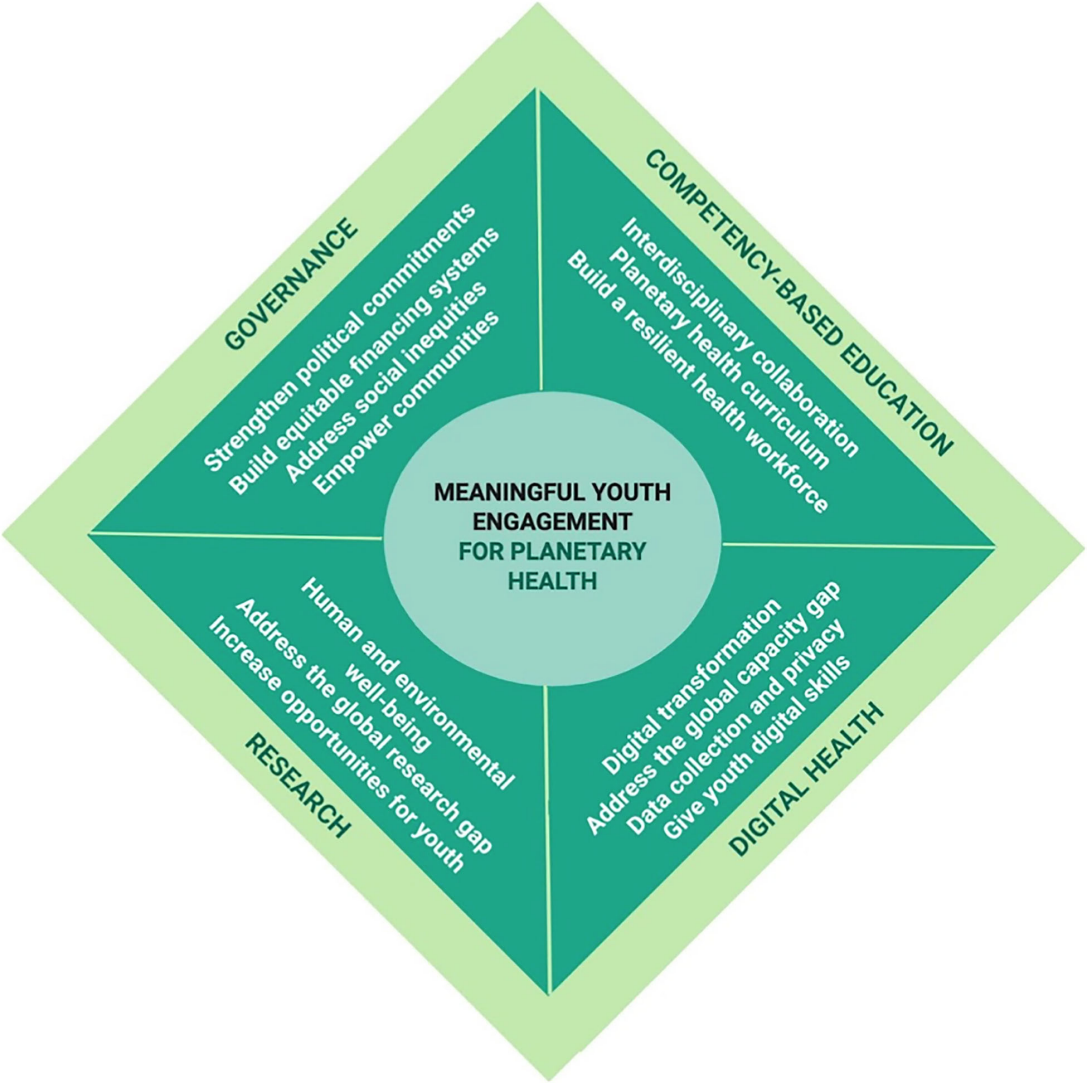
Youth‐inclusive planetary health framework. The framework is created based on the proceedings of the Global Health Workforce Network (GHWN) Youth Hub Virtual Conference 2022.

Based on these considerations, we created a youth‐inclusive planetary health framework (Figure 3). This framework presents the role and opportunities of meaningfully engaging youth in each planetary health pillar. Youth are centrally positioned in the diagram where they interact through key action areas with the four pillars (governance, competency‐based education, research, and digital health). There are no borders between the four sides of the rectangle as each pillar must also interact with each other when working toward better planetary health futures with youth. We will explain the means of youth engagement across the four pillars in the following sections.

### Governance

Good governance is important in tackling the immense challenges related to health workforce [8]. In the field of planetary health, cross‐sectoral governance is particularly important. Environmental policies should be created while considering the health lens of the issues and vice versa. This approach is exemplified by the Population, Equity, AIDS, and Coastal Environment (PEACE), a Tanzanian project launched in 2004. The project targeted HIV/AIDS‐affected households, which were found to depend more on coastal resources to survive HIV‐related poverty. Based on this condition, the PEACE project educated targeted communities to do farming and culture, both less threatening to the coastal diversity. Health care workers were also equipped with the knowledge to care for these families [18]. Another example is the Finnish Allergy Programme (2008–2018), where nature‐based approaches, including campaigns on reducing air pollution and smoking, led to a major reduction of asthma and allergy burdens in the country [19].

Scaling similar planetary health initiatives in national and/or global contexts requires strong political commitments and financing mechanisms. This can be conducted by first assessing the context of national and regional health systems, including identifying barriers and enablers to planetary health operationalization [11, 12].

Planetary health governance must also address social inequities. Effective planetary health governance can lessen the gap between low‐ and high‐income countries. Furthermore, vulnerable population groups, including children, youth, and women, must be supported to ensure planetary health operationalization and climate‐resilience across health systems globally [11, 20]. Community empowerment is crucial in addressing these inequities as involving grassroots movements for planetary health will allow for more sustainable culture‐sensitive approaches. For example, community health workers (CHWs) can be mobilized in planetary health programs, as is practiced in Indonesia, Brazil, and India [21, 22]. The Indonesian CHWs program includes CHWs not only in their human health systems but also in veterinary health programs [22].

Community empowerment should also involve the strategic and meaningful integration of youth voices. Including youth in everyday health governing processes can be achieved in multiple ways. They can carry the role of consultants and be included via various youth‐led networks. Governments can start giving regular visibility to youth engagement initiatives and establish institutional accountability mechanisms for ensuring continuous engagement with young people [23]. Table 1 shows several examples of youth participation in planetary health governance.

**TABLE 1:** Youth participation in global planetary health governance.

Involved entities	Youth participation
COP27	The COP27 presidency appointed a health care worker as the first youth envoy of the COP meetings. She aims to advocate for improving youth inclusion for better planetary health and climate resilience in global health systems [24]
UN Secretary General	The Youth Advisory Group on Climate Change provides advices and recommendations to the UN Secretary General [25]
Colombia	In 2018, the Supreme Court of Colombia ruled in favor of 25 youth regarding youth protection from deforestation and its impacts, including on health and safe water [26]
Indonesia	Youth is involved in the Climate Village program initiated by the Ministry of Environment and Forestry [17]
Kenya	The Ministry of Public Service, Youth, and Gender has consulted with young people during the drafting of national climate policies [17]

### Competency‐based education

Competency‐based education is crucial for promoting planetary health approaches among the health workforce. This will support their leadership role in addressing social inequalities and building collective capacity to ensure sustainability for human and planetary health [27]. Competencies, such as systems thinking training for young health professionals, have been shown to motivate more structured approaches to cross‐department communication and collaboration in pandemics response [28]. Youth possess a marked ability to process fresh ideas from interdisciplinary collaboration with a tenacious curiosity to address present and future challenges for planetary health.

The integration of planetary health into global health care education is currently underway. In 2021, The Planetary Health Alliance published the Planetary Health Education Framework, thus setting the scene for guiding the education of global citizens, practitioners, and professionals able and willing to address complex planetary health challenges. The framework included six key domains: interconnection within nature, the Anthropocene and health, equity and social justice, systems thinking and complexity, movement building, and systems change [13].

As of 2022, universities are making good progress in incorporating these domains into their curricula. According to the 2022 Planetary Health Report Card report, planetary health has been included to the curricula of 74 medical schools across the globe [29]. Some universities offer online short courses on planetary health that can be accessed by the public [27]. However, planetary health education is still lacking in institutions training allied health professionals [21]. Furthermore, the majority of available planetary health courses and curricula are developed by high‐income countries, thus potentially less inclusive for low‐income countries. Language barriers are still an issue even for online courses, as most are taught in English and German [27]. More investments in planetary health education will be beneficial to lessen this gap and ensure the sustainability of planetary health implementation.

### Research

Evidence‐based policies are known to be driven by research. The Rockefeller Foundation–Lancet Commission on Planetary Health puts research as one of the priority areas to improve planetary health. Planetary health research calls for urgent attention toward the human health cost of environmental degradation and invites more profound reflection on the relationship between human and environmental well‐being. It is not an easy task to research planetary health because the concept is new, gaps are wide, and funds are scarce [6].

Despite these barriers, planetary health research is growing in number. The Planetary Health Alliance features over 1200 articles covering various planetary health thematic areas [30]. A bibliometric analysis on climate and health research reported that 4247 articles were published between 1980 and 2019, with accelerated growth from 2010. However, the research production gap remains between lower‐ and higher‐income countries: The field is largely dominated by European countries and the United States, which represent 38.3% and 29.1% of the global research output, respectively. Only two lower‐middle‐income countries are among the top ten most active countries: China (5.5%) and India (3.2%) [31]. Global governments should provide more investments and efforts to lessen this gap.

Youth engagement is potentially the key to improving planetary health research globally. However, there is a lack of youth engagement in health research. A review highlighted that out of 420 studies collecting data from youth, just 21 included youth in the research process [32]. Health care training institutions need to increase opportunities for their students to conduct research in planetary health and collaborate with researchers in the field.

### Digital health

Digital health is transforming health systems globally. According to the IQVIA Institute for Human Data Science, there are approximately 350,000 digital health applications serving human health. About 90,000 of these applications were launched in 2020, averaging 250 applications per day [33]. Various platforms have also been developed to advance other aspects of planetary health, for example, environmental surveillance technologies that track the origins and causes of emerging outbreaks and disease‐related trends [34]. Digital health has a huge potential considering the widespread usage of digital technologies: 78% and 63.1% of the global population use smartphones and the Internet, respectively [35, 36]. Digital health platforms have lessened the gap in access to health care by connecting remote areas to the health system, as well as reducing the associated costs of seeking health care [33].

To maximize the impact of digital health on global health care systems, it is crucial to consider holistic approaches. However, the integration of planetary health approaches to digital health strategies is still limited. From our literature search, we found one proposed holistic approach to digital health called the One Digital Health Framework. The framework integrates One Health domains (human, animal, and environmental health) with digitalization. The framework proposes several functions of digital health utilization for One Health: (1) health care delivery in a syndemic scenario; (2) digital transformation of human and animal health data; and (3) digital nature conservation, such as biodiversity conservation and surveillance on food security, antimicrobrial resistance, and climate change, among others [34].

The digitalization of planetary health approaches will further improve the holistic nature of One Digital Health, as the concept also emphasizes social health. In general, planetary health digitalization can follow currently available digital health strategies globally, albeit with more considerations of animal, environmental, and social health. The process should also consider several dimensions identified by the One Digital Health Framework: The development of integrated and interconnected systems, which include real‐time data collection, digital health education, and community engagement [34]. Furthermore, the global capacity gap should be considered as local infrastructure capabilities, varying digital capacities of health care workers, and different global data privacy standards can pose a barrier to digital health collaboration and adoption [33]. Hence, global cooperation is needed to expedite knowledge transfer to scale the reinforcing impact of ideas.

Youth, as digital natives (generations born with influences from the internet and digital media), have huge potential to address these dimensions. Approximately 71% of youth use the internet, compared to 57% of the other age groups [37]. This digital experience, coupled with their eagerness to volunteer and support global change, provides opportunities for innovation. Equipping youth with the necessary digital health skills will allow them to transform the planetary health digitalization landscape, both in terms of education strategies and digital platform innovations. An example includes MyH2O, a youth‐led company in rural China aiming to provide clear water. The company employs a nationwide team of youth volunteers from various fields, including science and medicine, to gather real‐time data about local water sources and purification opportunities [38].

## RECOMMENDATIONS

Our conference called for a clearer understanding of enablers and barriers in planetary health operationalization to motivate greater levels of investment. This will require evidence from rigorous assessments conducted by close cooperation between global governments and international bodies. The impact of such work can be compounded through government partnerships with local universities and training programs to provide planetary health education to the future health workforce. Investments in planetary health research and digitalization are crucial to creating evidence‐informed strategies that reach wider communities. Governments should also collaborate and conduct knowledge transfer programs to lessen the gap in planetary health implementation between high‐ and low‐income countries.

Our conference and the resulting framework have highlighted the unique value of youth in planetary health and underscored the necessity of their inclusion in global action. Key assets lie in the significant size of the global youth community and their moral tenacity to build strong movements and coalitions for advocacy. Further, their digital adeptness, curious mindsets, and appetite for interdisciplinary collaboration position them to coalesce effective and inclusive solutions. Meaningful youth engagement in planetary health includes active involvement across governance, research, education, and digital health, alongside opportunities to share their knowledge and advice via advocacy.

Other youth‐led actions can be the benchmark of effective planetary health actions. Youth‐led planetary health actions should have a strong collaboration with senior professionals and stakeholders. This is exemplified by a WHO‐launched, youth‐led tuberculosis awareness project called the WHO 1+1 Initiative. The project has engaged and gained support from various stakeholders in Indonesia and Bangladesh [39]. The COVID‐19 pandemic has shown that interprofessional collaboration, as highlighted by our framework, is also crucial. Specifically, the collaboration will allow youth to utilize and combine the potentials of their unique skill sets and perspectives to effectively engage the community [40]. Their digital skills and creativity can also be harnessed to create an original approach to community involvement, as exemplified by the experience of a youth‐led cancer prevention awareness program that held a virtual art exhibition [41]. Global reach can be attained by youth by utilizing the power of social media and the Internet, thus enabling more impact [40, 41].

However, meaningful engagement requires deeper exploration into the barriers youth face when trying to position themselves as stakeholders in the multidisciplinary and overwhelming planetary health realm. Their opinions are often delegitimized due to perceived gaps in education and their needs are not prioritized [23]. The youth‐inclusive public health framework presented in this paper was produced from a strengths‐based approach and has the potential to expand and encompass a needs‐based perspective. The motivations, pains, and gains of both youth and institutions alike can be incorporated for added practicality in retention and synergistic use of active youth engagement to avoid tokenistic outcomes.

## CONCLUSION

Planetary health is an emerging holistic approach that is key to effective responses to various global health issues. Considering this importance, the state of meaningful youth engagement in planetary health needs to be improved. Youths, as discussed at the GHWN Youth Hub Virtual Conference 2022, are keen in advancing planetary health with their unique and innovative perspectives. Our framework provides youth‐inclusive approaches across four supporting pillars of planetary health: governance, competency‐based education, research, and digital health. More meaningful youth engagement will allow for sustainable planetary health operationalization globally.

## AUTHOR CONTRIBUTIONS

*Conceptualization; data curation; formal analysis; visualization; writing—original draft; writing—review and editing*: Adriana Viola Miranda, Katherine Feng. *Supervision; data curation; formal analysis; visualization; writing—original draft; writing—review and editing*: Ave Põld. *Formal analysis; writing—original draft; writing—review and editing*: Muhammed Yaseen Tagari, Sherly Meilianti. *Formal analysis; writing—original draft*: Shahd Sharief, Elaine Tan Su Yin, Rahmot Afolabi.

## CONFLICT OF INTEREST STATEMENT

Adriana Viola Miranda is an editorial board member of the journal. She was excluded and blinded from all stages of the peer review of this manuscript.

## FUNDING INFORMATION

There is no funding for the development of this paper.

## ETHICS STATEMENT

This is a conference proceeding. There is no need for ethical approval.

## Data Availability

No database or primary data was used in writing the paper.
